# The Effect of Vulcanization Temperature on the Network Structure and Properties of TBAF-Functionalized BR/VMQ Blends for Mars Environment Applications

**DOI:** 10.3390/ma19183846

**Published:** 2026-09-10

**Authors:** Norbert Nizel, Dariusz M. Bieliński, Jakub Wręczycki, Magdalena Maciejewska, Rafał Anyszka

**Affiliations:** Institute of Polymer and Dye Technology, Faculty of Chemistry, Lodz University of Technology, Stefanowskiego 16, 90-537 Lodz, Poland; dariusz.bielinski@p.lodz.pl (D.M.B.); jakub.wreczycki@p.lodz.pl (J.W.); magdalena.maciejewska@p.lodz.pl (M.M.)

**Keywords:** butadiene rubber, silicone rubber, low-temperature vulcanization, tetra-n-butylammonium fluoride, crosslink structure, Mars

## Abstract

**Highlights:**

**What are the main findings?**

- TBAF accelerates sulfur vulcanization of BR/VMQ blends at 100–140 °C.
- TBAF curing yields highly elastic networks with up to 97% polysulfidic bonds.
- Vulcanizing at 100–120 °C suppresses VMQ crystallization by over 80%.

**What are the implications of the main findings?**

- Fluoride anion sulfur activation has the potential to reduce energy consumption of rubber processing.
- TBAF-aided BR/VMQ composites provide stable dynamic performance in the Martian climate.
- Polysulfidic-dominated crosslink networks improve elasticity at low daily temperatures on Mars.
- Suppressed VMQ crystallization stabilizes the elastic modulus in Mars conditions.

**Abstract:**

Rubber compounds intended for Mars exploration missions must remain elastic at extremely low temperatures while being manufactured in a reliable and energy-efficient manner. In this study, the effect of vulcanization temperature (100–160 °C) on the network structure and properties of butadiene/silicone rubber (BR/VMQ) blends was investigated, comparing a conventional sulfur curing system (REF) with the same system activated by fluoride anion obtained from tetra-n-butylammonium fluoride (TBAF). The fluoride anion acts as an in situ activator of elemental sulfur, through the opening of the S_8_ ring, facilitating the crosslinking process at significantly lower temperatures. Fluoride enabled rapid vulcanization at 100–120 °C, shortening the optimum cure time from 129.1 min to 61.4 min at 100 °C and from 36.7 min to 16.9 min at 120 °C. Equilibrium swelling and thiol-amine analysis revealed opposite structural responses to lowered curing temperature: the crosslink density of the reference compounds increased (from 1.25 × 10^−4^ to 1.56 × 10^−4^ mol/cm^3^ between 160 °C and 120 °C), whereas that of the TBAF-containing compounds decreased (from 1.32 × 10^−4^ to 0.60 × 10^−4^ mol/cm^3^ between 160 °C and 100 °C), yielding networks dominated by elastic polysulfidic crosslinks (up to 97.2%). We attribute this to a suppressed crosslink maturation under conditions of reduced thermal energy and shortened curing time. Low-temperature curing also suppressed the crystallization of the VMQ phase (melting enthalpy decreasing from 1.23 J/g to 0.21 J/g for TBAF compounds), which we hypothesize results from insufficient energy for phase separation and regular chain packing in this strongly immiscible blend. TBAF-cured compounds exhibited lower tanδ peaks, a stable tanδ plateau between approximately −60 °C and +20 °C, a tanδ-peak shift towards lower temperatures with decreasing curing temperature, and higher elongation at break and tensile strength at −40 °C. The results show that low-temperature, fluoride-activated vulcanization is a promising route for tailoring BR/VMQ networks towards stable dynamic performance across the Martian daily temperature range.

## 1. Introduction

Designing elastomer composites capable of maintaining low-temperature flexibility without embrittlement remains a significant challenge in materials engineering. This challenge is even greater in space applications, such as the exploration of the Martian surface, where materials can be subjected to extreme temperature cycles ranging from +30 °C down to −120 °C. So far, all Mars rovers have driven on wheels made of aluminum alloys, which—as shown by the reports of Curiosity wheel damage—are vulnerable to long-term dynamic fatigue [1,2]. To overcome the limitations of rigid metals, transitioning to elastomeric components is highly desirable; however, this comes with strict mechanical requirements. For example, elastomeric rover tires must be capable of absorbing severe impact energy from constant contact with sharp rocks without shattering at deep sub-zero temperatures. Similarly, external dynamic seals and flexible joints must maintain a constant sealing force and a stable spring rate across daily temperature changes to prevent atmospheric leaks. Therefore, the critical requirement for any Martian elastomer is not only a low glass transition temperature, but a highly elastic, energy-dissipating network architecture capable of withstanding large mechanical deformations throughout wide temperature amplitudes.

In our previous research, we introduced blends of butadiene rubber (BR) and vinylmethyl silicone rubber (VMQ), selected for their very low glass transition temperatures (Tg ≈ −90 °C and −129 °C, respectively). We demonstrated their viable mechanical properties, outstanding gamma-radiation resistance, and consistent low-temperature dynamic behavior when the amorphous BR is used [3,4,5,6,7]. However, the two rubbers are thermodynamically immiscible, forming a two-phase morphology with VMQ dispersed in a continuous BR matrix. Despite this, our study demonstrated that Carbon Black exhibits good interfacial interactions with both the BR and VMQ phases, effectively acting as a solid compatibilizer [8]. While standard silicone rubbers cannot be crosslinked by sulfur, the specific VMQ grade selected for this blend contains a relatively high amount of vinyl groups (1%), which allows it to react with sulfur-based curatives and co-vulcanize with the BR matrix [8]. Because of this physical and chemical coupling, the processing route—particularly the vulcanization step—may be expected to influence both the crosslink network and the phase behavior of such blends.

Vulcanization is conventionally carried out at 140–200 °C and constitutes one of the most energy-intensive steps of rubber processing. There is a general belief among rubber industry experts that the conventional tire vulcanization process is dramatically inefficient, and recent modeling combined with direct measurements confirms this, indicating that the industry urgently needs more efficient heating strategies [9]. Reducing the vulcanization temperature is therefore positive from both economic and environmental perspectives. Moreover, it has been found that lowering the vulcanization temperature can enhance scorch safety and mechanical properties, although curing then takes a longer time, which may not be economically favorable [10]. Accordingly, recent efforts have focused on curing systems that accelerate vulcanization at lower temperatures, e.g., MgO/ZnO co-activator systems [10]. An alternative, chemically distinct strategy is the nucleophilic activation of elemental sulfur. The activation of the S_8_ ring by its opening under the action of various nucleophiles is considered a promising green-chemistry alternative to the energy-intensive thermal generation of sulfur diradicals [11]. In organic synthesis, the fluoride anion was demonstrated to be a superior activator of elemental sulfur, generating in situ reactive, nucleophilic fluoropolysulfide anions FS_(8−x)_^−^ [12], and elemental sulfur undergoes such activation upon treatment with catalytic amounts of tetra-n-butylammonium fluoride (TBAF) even at room temperature [13].

Recently, we demonstrated that the incorporation of a small amount of TBAF into a conventional sulfur/CBS curing system drastically reduces the temperature required for vulcanization [14,15]. This raises two questions relevant to the Mars-oriented rubber: (i) how does lowering the vulcanization temperature affect the crosslink density and crosslink architecture of BR/VMQ blends, and (ii) do these structural changes translate into the low-temperature static and dynamic properties that define the material’s usefulness in the Martian environment, where the daily temperature amplitude can reach 100 °C [3,4]? The type of sulfidic crosslink is known to be decisive here: vulcanizates with predominantly polysulfidic crosslinks exhibit tensile strength, modulus, and elongation at break greater than those of the corresponding monosulfidic networks [16], since monosulfidic crosslinks cannot exchange, rearrange, or break to relieve stresses without main-chain scission, whereas polysulfidic crosslinks are able to rearrange under stress [17].

In this paper, BR/VMQ compounds with and without TBAF were vulcanized at 160 °C, 140 °C, 120 °C, and 100 °C. Vulcanization characteristics, crosslink density and structure (equilibrium swelling and thiol-amine analysis), static mechanical properties at room temperature and at −40 °C, dynamic mechanical properties, and thermal transitions (DSC) were investigated to establish the relationship between curing temperature, network architecture, and the low-temperature performance of the blends.

## 2. Materials and Methods

### 2.1. Materials

Medium-cis lithium BR, Buna^®^ CB550, was provided by Arlanxeo, Geleen, The Netherlands. Vinyl methyl silicone rubber (VMQ) containing one vinyl group per 99 methyl groups in the polymer backbone (Polimer^®^ MV 1,0) was provided by Silikony Polskie, Nowa Sarzyna, Poland. Rubber additives—activators: zinc oxide, stearic acid; accelerator: N-cyclohexyl-2-benzothiazole sulfenamide (CBS); curative: sulfur; antiozonant: N-(1,3-dimethylbutyl)-N’-phenyl-p-phenylenediamine (6PPD)—were supplied by Torimex Chemicals, Lodz, Poland. Carbon black (CB) N330 grade (BET surface area of 78 m^2^/g) was provided by Birla Carbon, Trecate, Italy. Tetra-n-butylammonium fluoride (TBAF) trihydrate was purchased from Merck KGaA, Darmstadt, Germany. Toluene (analytical grade) used for equilibrium swelling was purchased from Chempur, Piekary Śląskie, Poland. Reagents used for thiol-amine analysis were purchased from Merck KGaA, Darmstadt, Germany (Piperidine, 99%) and Acros Organics, Geel, Belgium (propane-2-thiol, 98%; dodecane-1-thiol, 98%).

### 2.2. Elastomer Blends Preparation

Elastomer blends were prepared using a laboratory mixer Plastograph^®^ Lab-Station (Brabender, Germany) with the mixing chamber heated to 70 °C, following the formulations presented in Table 1 and the mixing procedure described in Table 2. The use of a BR/VMQ ratio of 80/20 is explained in our previous research [7]. Two compounds were prepared: a reference (REF) and a TBAF-modified compound (TBAF), differing only in the addition of TBAF. The amount of tetra-n-butylammonium fluoride used in the rubber mixtures was calculated as 50 mol% amount of sulfur used, which corresponded to 0.74 phr (this was found to be the recommended value for the diene rubbers in our patent research [14]).

### 2.3. Vulcanization Characteristics

Vulcanization curves were obtained using an MDR 2000 rheometer (Alpha Technologies, Hudson, OH, USA). From the curves, the optimum cure time (time to reach 90% of ΔM, t_90_), scorch time (time to reach 5% of ΔM, t_05_), minimum torque (ML), maximum torque (MH), and torque increase (ΔM = MH − ML) were obtained. The measurements were carried out at various temperatures with correspondingly extended test times to fully capture the curing process at lower temperatures: 160 °C (20 min), 140 °C (40 min), 120 °C (40 min for TBAF-modified samples, 120 min for the reference), and 100 °C (120 min for TBAF-modified samples, 180 min for the reference). Additionally, the cure rate index was calculated as CRI = 100/(t_90_ − t_05_). To support the characteristics investigation, uncured compounds were analyzed by DSC to record the curing exotherm (according to the methodology described in Section 2.9).

### 2.4. Vulcanization

Flat sheet vulcanizates were prepared using steel molds and an in-house (custom-built) laboratory hydraulic press at 160 °C, 140 °C, 120 °C, and 100 °C under 10 MPa of pressure. The vulcanization time of each sample was equal to the optimum cure time of the corresponding compound at the given temperature (determined via rheometric measurements, as detailed in Section 3.1). Samples are hereafter denoted by the compound type and curing temperature (e.g., TBAF 120).

### 2.5. Crosslink Density

Crosslink density was determined through equilibrium swelling in toluene using the Flory–Rehner equation [18]. Vulcanizate samples of 30–50 mg were submerged in toluene and left for 72 h at room temperature. The swollen samples were then weighed and dried in a laboratory oven at 70 °C for 72 h, after which the dry samples were weighed. The crosslink density ν [mol/cm^3^] was calculated as shown in Equation (1),

(1) $$\upsilon =-\frac{\ln\left(1-V_{r}\right)+V_{r}+\chi V_{r}^{2}}{V_{0}\cdot \left(V_{r}^{\frac{1}{3}}-\frac{2V_{r}}{f}\right)}$$

where V_r_ is the volume fraction of rubber in the swollen sample, V_0_ the molar volume of the solvent, f the crosslink functionality (f = 4, assuming tetrafunctional crosslinks as the standard topological assumption for conventional sulfur vulcanization networks), and χ the Flory–Huggins polymer–solvent interaction parameter. The parameter χ was calculated using the Bristow–Watson equation [19] (Equation (2)):

(2) $$\chi_{12}=\frac{\left[V{\left(\delta_{1}-\delta_{2}\right)}^{2}\right]}{RT}+0.34$$

where V is the molar volume of the solvent (106.3 cm^3^/mol for toluene), R is the universal gas constant, T is the temperature (in Kelvin), and δ_1_, δ_2_ are the Hildebrand solubility parameters of the solvent and the polymer.

The following literature solubility parameters were adopted:

δ_(toluene)_ = 18.2 MPa^1/2, δ_(BR)_ = 17.1 MPa^1/2 [20], and δ_(VMQ)_ = 15.5 MPa^1/2 [21].

This yields χ_(BR–toluene)_ ≈ 0.39 and χ_(VMQ–toluene)_ ≈ 0.65; to obtain an effective interaction parameter of the immiscible blend, the material was treated mathematically as a pseudo-homogeneous system and χ ≈ 0.44 was estimated by weighting the component values with the rubber volume fractions (0.8/0.2). A similar approach to calculating the Flory–Huggins interaction parameter was previously reported by Abdelsalam et al. for ternary rubber blends of NR/SBR/NBR [22].

The volume fraction of rubber V_r_ was obtained from Equation (3):

(3) $$V_{r}=\frac{1}{1+Q_{v}}$$

where Q_v_—equilibrium volume swelling, obtained from Equation (4):

(4) $$Q_{v}=Q_{w}\cdot \frac{\rho_{r}}{\rho_{s}}$$

where

Q_w_—equilibrium mass swelling, ρ_r_—density of the rubber (ρ_BR/VMQ_ = 0.968 g/cm^3^), ρ_s_—density of the solvent (ρ_toluene_ = 0.8669 g/cm^3^).

Q_w_ was determined using Equation (5):

(5) $$Q_{w}=\frac{m_{s}-m_{d}}{m_{d}-m_{0}f’}$$

where

m_s_—sample mass in swollen state, m_d_—dried sample mass, m_0_—sample initial mass, f′—fraction of insoluble particles (filler + ZnO).

For each vulcanizate, equilibrium swelling measurements were performed on four independent specimens (n=4), and the calculated crosslink densities are reported as the mean value.

### 2.6. Crosslink Structure

The crosslink structure was determined using thiol-amine analysis. To selectively cleave di- and polysulfidic bonds, 200–250 mg samples were placed in a 1 mol/L solution of dodecanethiol in piperidine for 24 h under a nitrogen atmosphere. To selectively cleave polysulfidic bonds, 200–250 mg samples were placed in a solution of 0.4 mol/L propane-2-thiol and 0.4 mol/L piperidine in toluene for 2 h. Afterwards, all samples were washed with toluene until the remains of the thiol-amine solutions were removed, cut into four pieces, and subjected to the swelling procedure described in Section 2.5. Because m_0_ could not be measured for the treated samples, the equilibrium mass swelling was obtained as follows:

(6) $$Q_{w}=\frac{m_{s}\;-\;m_{d}}{m_{d}(1\;-\;f’)}.$$

It has to be noted that samples REF 140, REF 100, TBAF 140, TBAF 120, and TBAF 100 crumbled after the selective degradation of di- and polysulfidic bonds, which itself indicates that their networks contain a very low content of C–C and monosulfidic crosslinks and that the macroscopic integrity of the network relied entirely on the cleaved di- and polysulfidic crosslinks.

### 2.7. Static Mechanical Properties at Room Temperature and at −40 °C

Stress–strain curves were determined using a Universal Testing Machine Z005 equipped with a thermostatically controlled chamber (ZwickRoell, Ulm, Germany). For sub-zero measurements, the chamber was cooled with liquid nitrogen to −40 °C. The tests were performed in accordance with the ISO 37 [23] standard using type 2 dumbbell-shaped test pieces cut from the flat-sheet vulcanizates. Representative curves are presented; the maximum elongation of the testing apparatus was 1000%, and for samples that did not break, the stress recorded at maximum elongation (T_Fmax_) is reported instead of tensile strength.

### 2.8. Dynamic Mechanical Analysis

Storage modulus E′, loss modulus E″, and tanδ values were recorded in the temperature range from −145 °C to 30 °C using a DMA1 Dynamic Mechanical Analyzer (Mettler Toledo, Columbus, OH, USA). Samples of rectangular cross-section (4 mm × 2 mm), obtained from the type 2 dumbbell-shaped test pieces (ISO 37), were used for testing. The test was performed using tension mode with a heating rate of 2 K/min and a frequency of 10 Hz.

### 2.9. Differential Scanning Calorimetry

Differential scanning calorimetry (DSC) was performed using a DSC3 calorimeter (Mettler Toledo, Columbus, OH, USA). Heat flow was measured in the temperature range from −150 °C to 30 °C in a nitrogen atmosphere at a heating/cooling rate of 20 K/min. DSC was performed only on samples cured at the boundary temperatures (160 °C and 100 °C) and at 120 °C, identified as the most promising low-temperature condition; the 140 °C samples were omitted to optimize analytical resources; testing focused strictly on the boundary temperatures (160 °C and 100 °C) and the 120 °C condition, which was preemptively identified as the optimal low-temperature vulcanization point based on prior patent research [14].

Additionally, DSC of uncured REF and TBAF compounds was performed in the range from 0 °C to 250 °C in a nitrogen atmosphere at a heating rate of 10 K/min.

## 3. Results and Discussion

### 3.1. Vulcanization Characteristics

**Figure 1:**
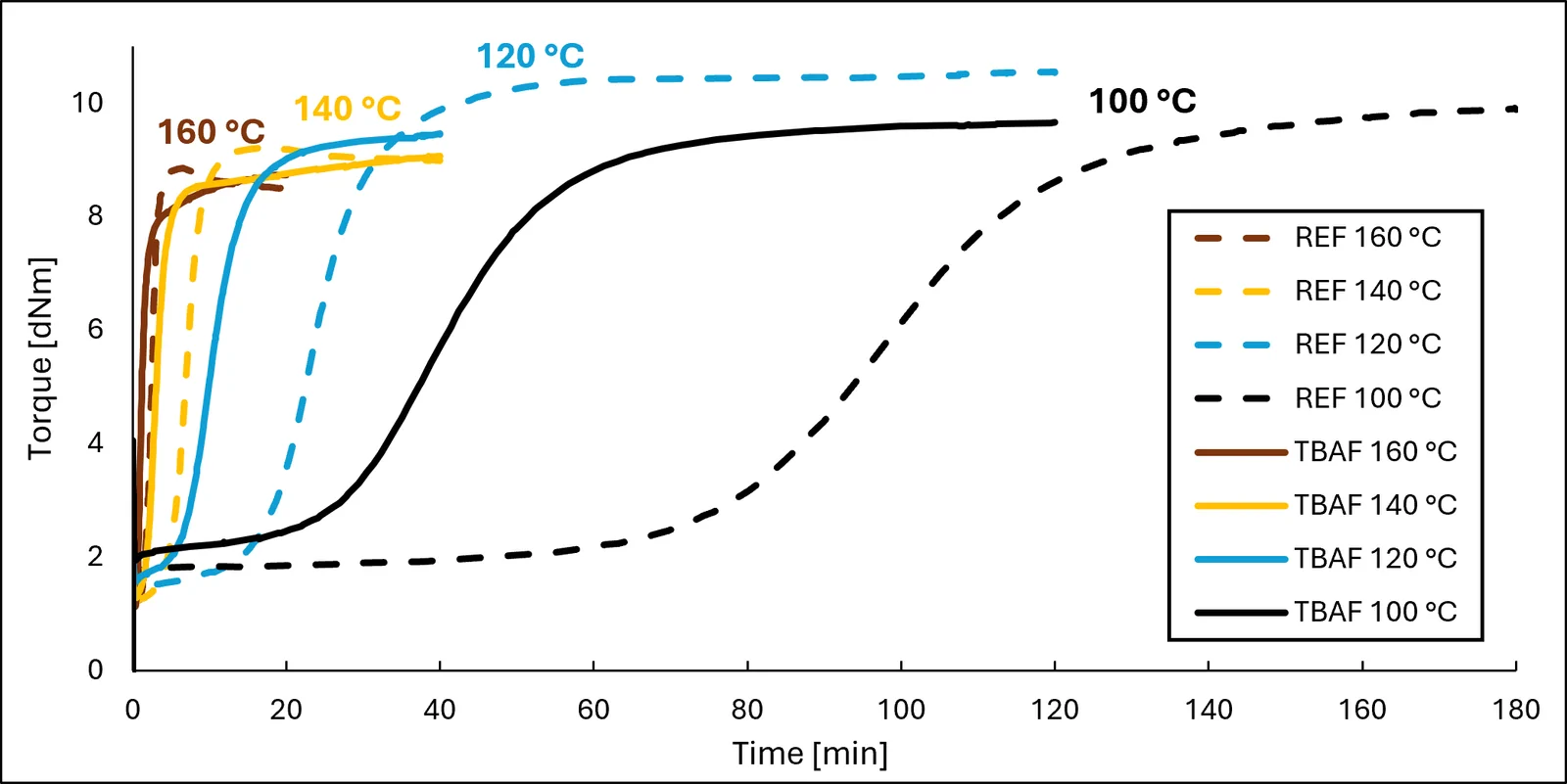
Vulcanization curves of BR/VMQ composites vulcanized at different temperatures in the presence (TBAF) or absence (REF) of fluoride anion in the compound.

The vulcanization curves of the REF and TBAF compounds at all four temperatures are shown in Figure 1, and the derived parameters are collected in Table 3.

**Table 3:** Minimum torque (ML), maximum torque (MH), torque increase (ΔM), scorch time (t_05_), optimum cure time (t_90_), and cure rate index (CRI) of the BR/VMQ compounds.

Sample	ML [dNm]	MH [dNm]	ΔM [dNm]	t_05_ [min]	t_90_ [min]	CRI [%/min]
REF 160	1.2	8.9	7.7	1.3	3.4	49.02
REF 140	1.2	9.2	8.0	3.6	9.4	17.27
REF 120	1.4	10.6	9.2	11.9	36.7	4.02
REF 100	1.7	9.9	8.2	57.6	129.1	1.40
TBAF 160	1.1	8.8	7.6	0.9	3.7	34.97
TBAF 140	1.3	9.1	7.8	1.5	6.1	21.51
TBAF 120	1.6	9.5	7.9	4.8	16.9	8.31
TBAF 100	2.0	9.7	7.7	16.4	61.4	2.22

At 160 °C, both compounds cure similarly (t_90_ of 3.4 min and 3.7 min), showing that at conventional temperatures the thermal opening of the S_8_ ring dominates and the contribution of the fluoride activation is marginal. The benefit of TBAF becomes pronounced as the temperature decreases: at 120 °C the optimum cure time is reduced from 36.7 min to 16.9 min, and at 100 °C from 129.1 min to 61.4 min (the CRI more than doubles at 120 °C and increases roughly 1.6-fold at 100 °C). The scorch time follows the same pattern (57.6 min vs. 16.4 min at 100 °C, but 3.6 min vs. 1.5 min at 140 °C) showing potential processing difficulties if the TBAF-mediated vulcanization is used at high curing temperatures. The DSC measurements of the uncured compounds (shown in Section 3.5) are highly consistent with this facilitated sulfur activation. The curing exotherm of the reference compound has an onset at 164.6 °C (peak at 171.3 °C), whereas for the TBAF compound the onset is shifted down to 133.8 °C (peak at 147.7 °C), representing a substantial reduction in the thermal threshold required to initiate vulcanization. Furthermore, the integrated enthalpy of the TBAF curing exotherm is noticeably lower (0.27 J/g) than that of the REF compound (1.47 J/g). This behavior is consistent with the proposed mechanism, in which nucleophilic fluoropolysulfide anions are generated in situ from S_8_ and the fluoride anion [12], bypassing the high-energy homolytic S–S cleavage and altering the overall heat of the curing reaction.

For both compounds, ML increases with decreasing curing temperature (from 1.2 to 1.7 dNm for REF and from 1.1 to 2.0 dNm for TBAF), which reflects the higher viscosity of the uncured compound at lower temperatures. More interesting is the behavior of MH. For reference, MH increases from 8.9 dNm (160 °C) to 10.6 dNm (120 °C), suggesting a denser network formed during the much longer cure. The REF 100 sample deviates from this trend (MH = 9.9 dNm); we hypothesize that this anomaly reflects the physical limits of the conventional curing system at this specific temperature, as 100 °C lies below the melting point of elemental sulfur (~115 °C), and incomplete dissolution of the curative in the rubber matrix cannot be excluded. For the TBAF compounds, MH increases only moderately (8.8 → 9.7 dNm), and at every temperature below 160 °C, the TBAF compound exhibits lower MH than its REF counterpart, indicating the differences in crosslink density discussed in the following section.

### 3.2. Crosslink Density and Architecture

**Figure 2:**
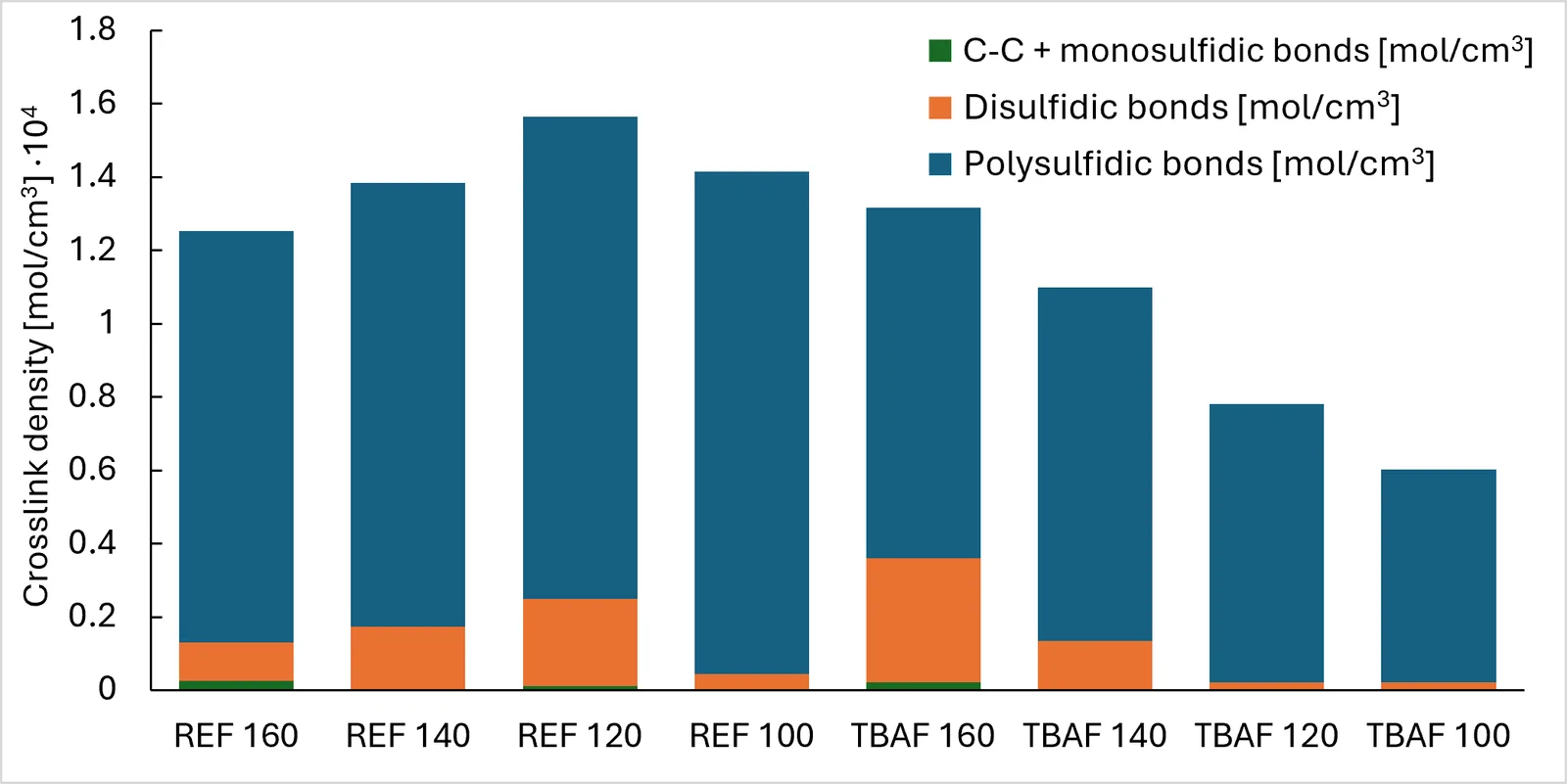
Crosslink density and structure of the BR/VMQ composites vulcanized at different temperatures in the presence (TBAF) or absence (REF) of fluoride anion in the compound.

The crosslink densities and crosslink structures obtained from equilibrium swelling and thiol-amine analysis are presented in Table 4 and Figure 2.

Two opposite trends emerge. For the reference compounds, lowering the curing temperature from 160 °C to 120 °C increases the crosslink density (1.25 → 1.56 × 10^−4^ mol/cm^3^) and decreases the polysulfidic fraction (89.6% → 84.1%) in favor of disulfidic bonds. Our hypothesis is that the drastically longer curing times at lower temperatures (t_90_ = 36.7 min at 120 °C vs. 3.4 min at 160 °C) give more time for crosslink maturation. In sulfur vulcanization, the initially formed polysulfidic bridges undergo slow desulfuration and rearrangement, whereby the liberated sulfur is re-inserted into the network as new, shorter crosslinks; the temperature dependence of the stability of such intermediates and crosslinks was described in detail by Morrison and Porter [24]. At 120 °C, the ten times longer period spent in the mold evidently compensates for the reduced thermal energy, so that maturation proceeds: polysulfidic bridges are shortened to disulfidic ones, and the released sulfur creates additional network junctions, raising the crosslink density. At 160 °C, in contrast, the cure is completed within minutes and is terminated before maturation can advance, while thermal reversion additionally limits the final crosslink density. The REF 100 sample again falls outside the trend (ν = 1.42 × 10^−4^ mol/cm^3^ at 96.9% polysulfidic crosslinks), which supports its classification as an anomaly—below the melting point of sulfur, locally curative-rich domains may form long polysulfidic bridges, while the network, as a whole, develops inhomogeneously.

The TBAF compounds behave in the opposite manner. Because the fluoride-activated pathway generates reactive polysulfide species without requiring high temperature, crosslink insertion at 120 °C and 100 °C is fast relative to maturation, and the reduced thermal energy together with the shortened optimum cure time effectively freezes the network in its unmatured state: long, sulfur-rich bridges are retained (97.2% and 96.5% polysulfidic crosslinks for TBAF 120 and TBAF 100, respectively), and since each polysulfidic bridge consumes several sulfur atoms, the same sulfur loading of 1.2 phr translates into fewer crosslinks overall—hence the monotonic decrease in crosslink density from 1.32 × 10^−4^ mol/cm^3^ (160 °C) to 0.60 × 10^−4^ mol/cm^3^ (100 °C). The crumbling of the TBAF 140, TBAF 120, and TBAF 100 samples after selective cleavage of di- and polysulfidic bonds shows that C–C and monosulfidic junctions are practically absent from these networks.

To contextualize the results of the thiol-amine analysis, the distinct mechanical and thermal roles of each linkage type must be highlighted. Monosulfidic (S_1_) and carbon-carbon (C–C) crosslinks are short and rigid; their inability to slip or rearrange under load concentrates local stress and typically leads to lower elongation at break [16]. Disulfidic (S_2_) linkages offer an intermediate balance of stability and flexibility. In contrast, polysulfidic linkages (S_x_, x ≥ 3) are long and highly flexible. Although they are more thermally labile, their extended structure should impose fewer constraints on polymer chain mobility and free volume, influencing the fundamental thermal and dynamic behavior of the elastomer [25]. More importantly for sub-zero applications, polysulfidic bridges can break, slip, and reform under mechanical stress [17], allowing the network to safely dissipate applied energy rather than rupturing.

From the application standpoint, this architecture is advantageous, as vulcanizates whose crosslinks are predominantly polysulfidic exhibit tensile strength, modulus, and elongation at break greater than those of the corresponding monosulfidic networks [16], owing to the ability of polysulfidic bridges to rearrange and dissipate local stresses without main-chain scission [17].

It must be acknowledged that in conventional ‘earthly’ applications (such as automotive tires), an immature network dominated by polysulfidic crosslinks is often susceptible to thermal reversion and ongoing maturation. Dynamic flexing, internal heat build-up, or even long-term room-temperature storage can provide the activation energy necessary for these S_x_ bonds to resume desulfuration, leading to undesired changes in mechanical properties over time. However, this problem is mostly avoided in the context of Martian exploration. Surface temperatures on Mars only rarely exceed +20 °C, with the vast majority of the rover’s operational life spent at extreme sub-zero temperatures (down to −120 °C). The thermal energy required to drive post-cure crosslink maturation is unlikely to be reached in the target environment. We hypothesize that the unique, highly elastic polysulfidic architecture generated by the TBAF-aided curing is thermally protected and potentially locked in its stress-dissipating state for the duration of the mission [24].

### 3.3. Static Mechanical Properties at Room Temperature and at −40 °C

**Figure 3:**
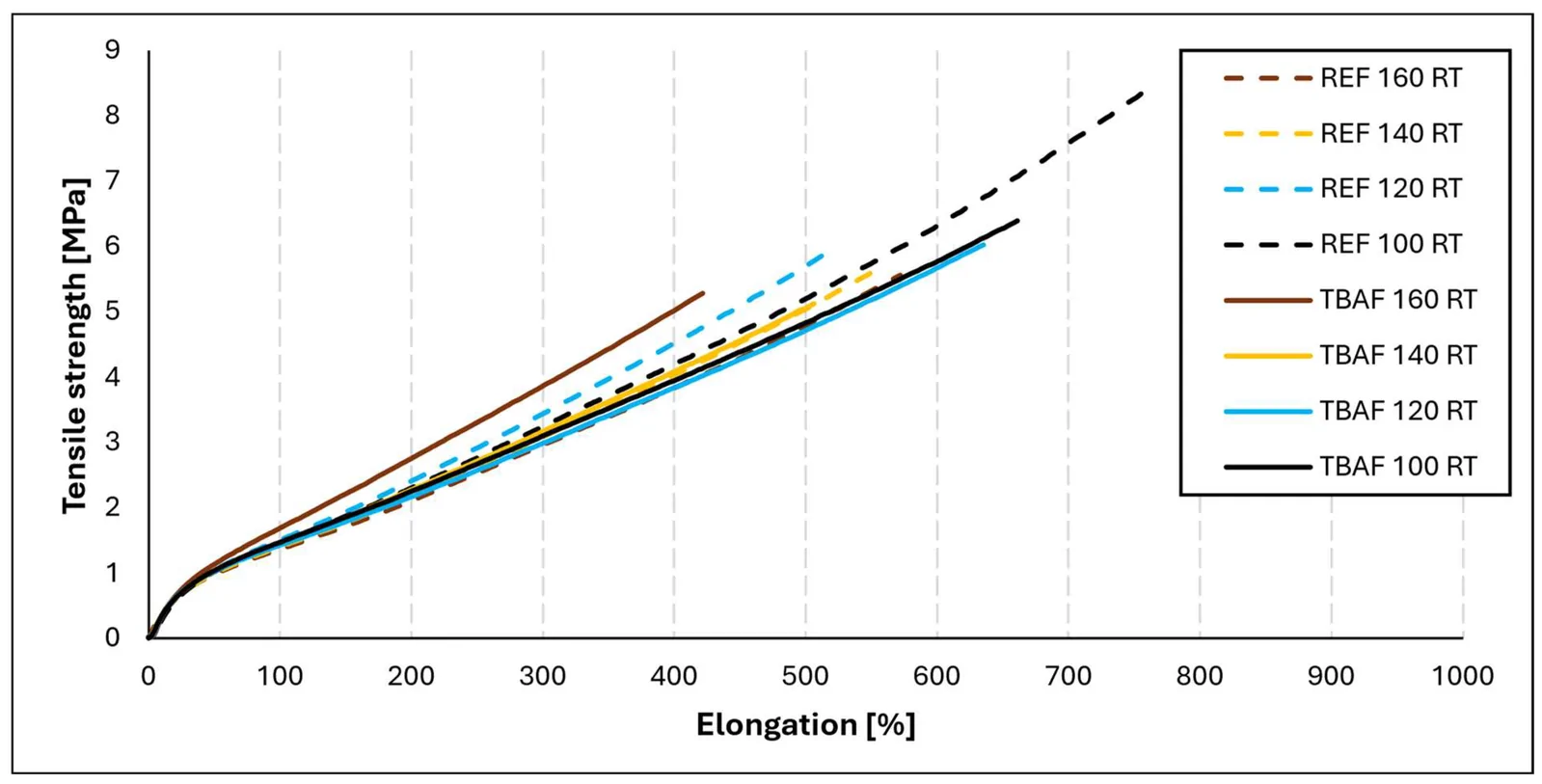
Stress–strain curves recorded at room temperature for BR/VMQ composites vulcanized at different temperatures in the presence (TBAF) or absence (REF) of fluoride anion in the compound.

**Figure 4:**
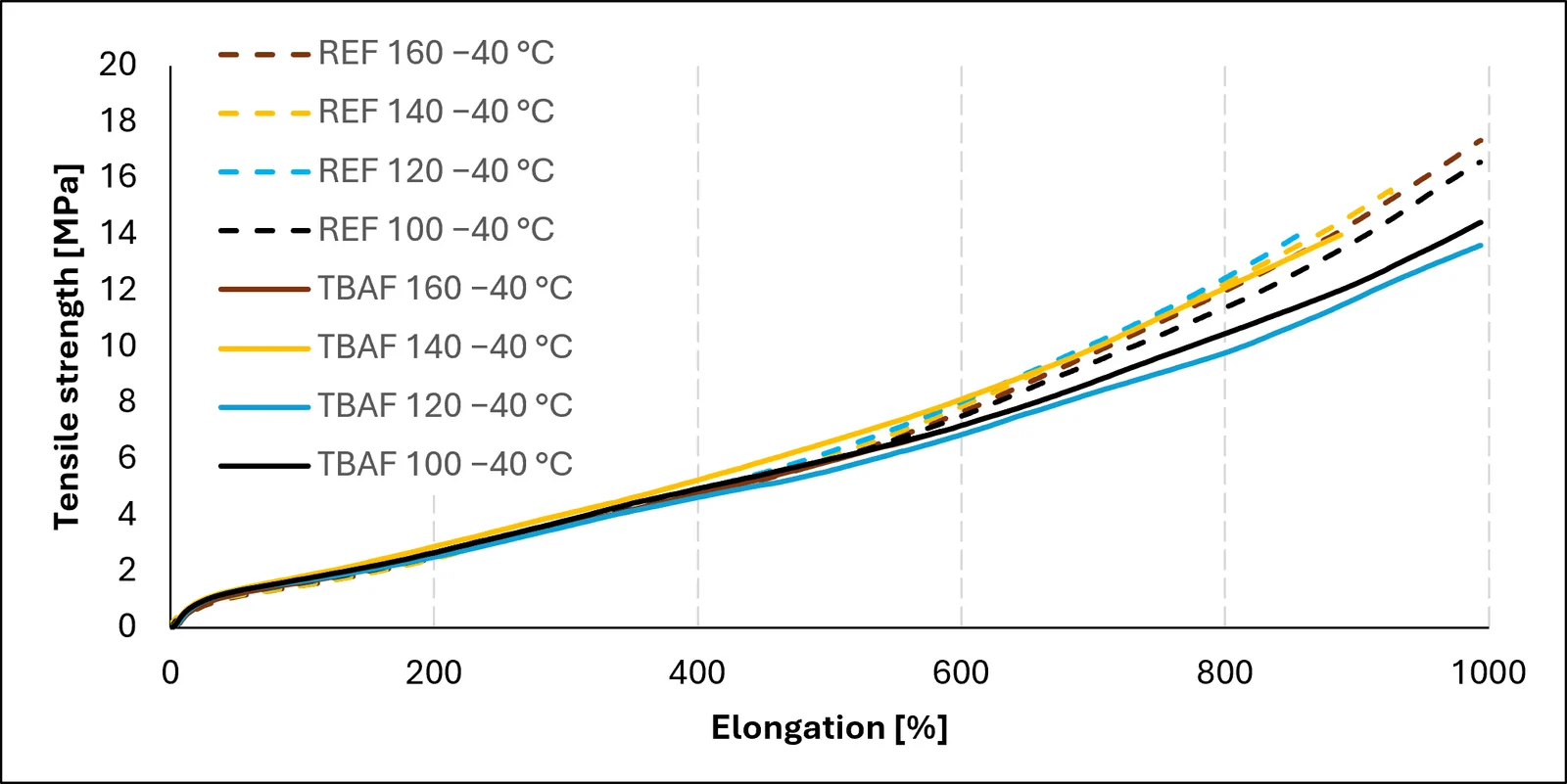
Stress–strain curves recorded at −40 °C for BR/VMQ composites vulcanized at different temperatures in the presence (TBAF) or absence (REF) of fluoride anion in the compound.

Representative stress–strain curves recorded at room temperature and at −40 °C are shown in Figure 3 and Figure 4. Tensile strength at break (T_S_) or stress at maximum elongation T_Fmax_ (indicated with †) and elongation at break (E_B_) values are presented in Table 5. At room temperature, the trends follow the crosslink densities discussed above: the reference vulcanizates cured at 140 °C and 120 °C, having the highest network density, exhibit the highest stiffness (highest stress at a given elongation), whereas the TBAF vulcanizates cured at 120 °C and 100 °C are the most extensible, several of them not breaking within the 1000% elongation limit of the testing setup (for these, T_Fmax_ is reported instead of tensile strength to distinctly differentiate it from the tensile strength at break). At −40 °C (an important testing temperature that aligns with both the melting/crystallization region of the VMQ phase and the typical daily thermal cycling on Mars), the differences become more pronounced: the TBAF vulcanizates cured at reduced temperatures retain higher extensibility and lower stress at corresponding elongations than their REF counterparts. We attribute this to the combination of (i) their lower crosslink density, (ii) their almost purely polysulfidic, stress-rearrangeable networks (unlike C-C or monosulfidic bonds, polysulfidic bonds can break, slip and reform under mechanical stress, allowing the network to dissipate energy rather than rupturing) [16,17], and (iii) the suppressed crystallinity of the VMQ phase, discussed in Section 3.5, which removes rigid crystalline domains that would otherwise act as stress concentrators just below their melting region (which was observed around −40 °C, as shown in Section 3.5.). The direction of these mechanical differences remains highly consistent across both test temperatures.

### 3.4. Dynamic Mechanical Analysis

**Figure 5:**
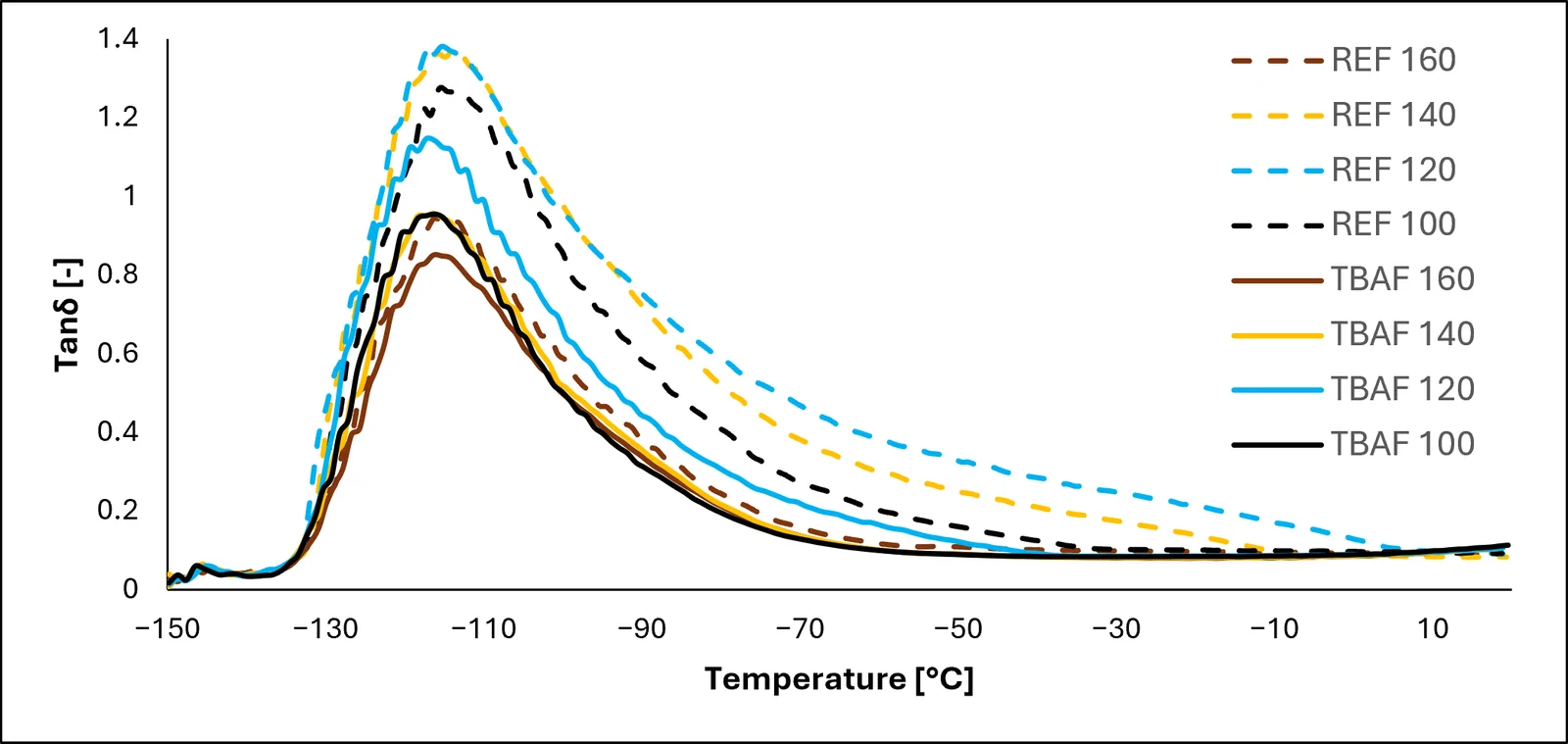
Tanδ curves of the BR/VMQ composites vulcanized at different temperatures in the presence (TBAF) or absence (REF) of fluoride anion in the compound.

**Figure 6:**
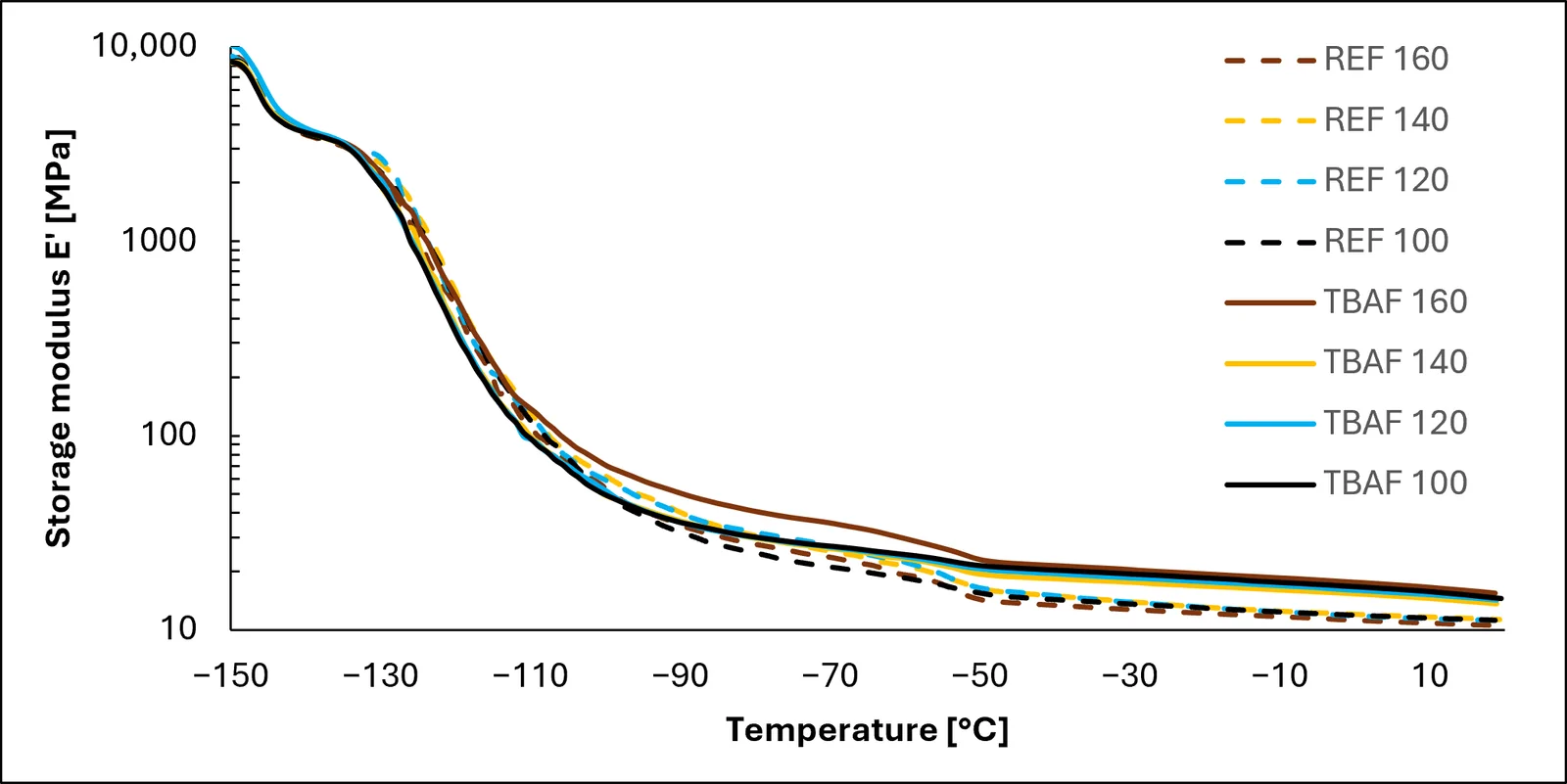
Storage modulus (E′) curves of the BR/VMQ composites vulcanized at different temperatures in the presence (TBAF) or absence (REF) of fluoride anion in the compound.

**Figure 7:**
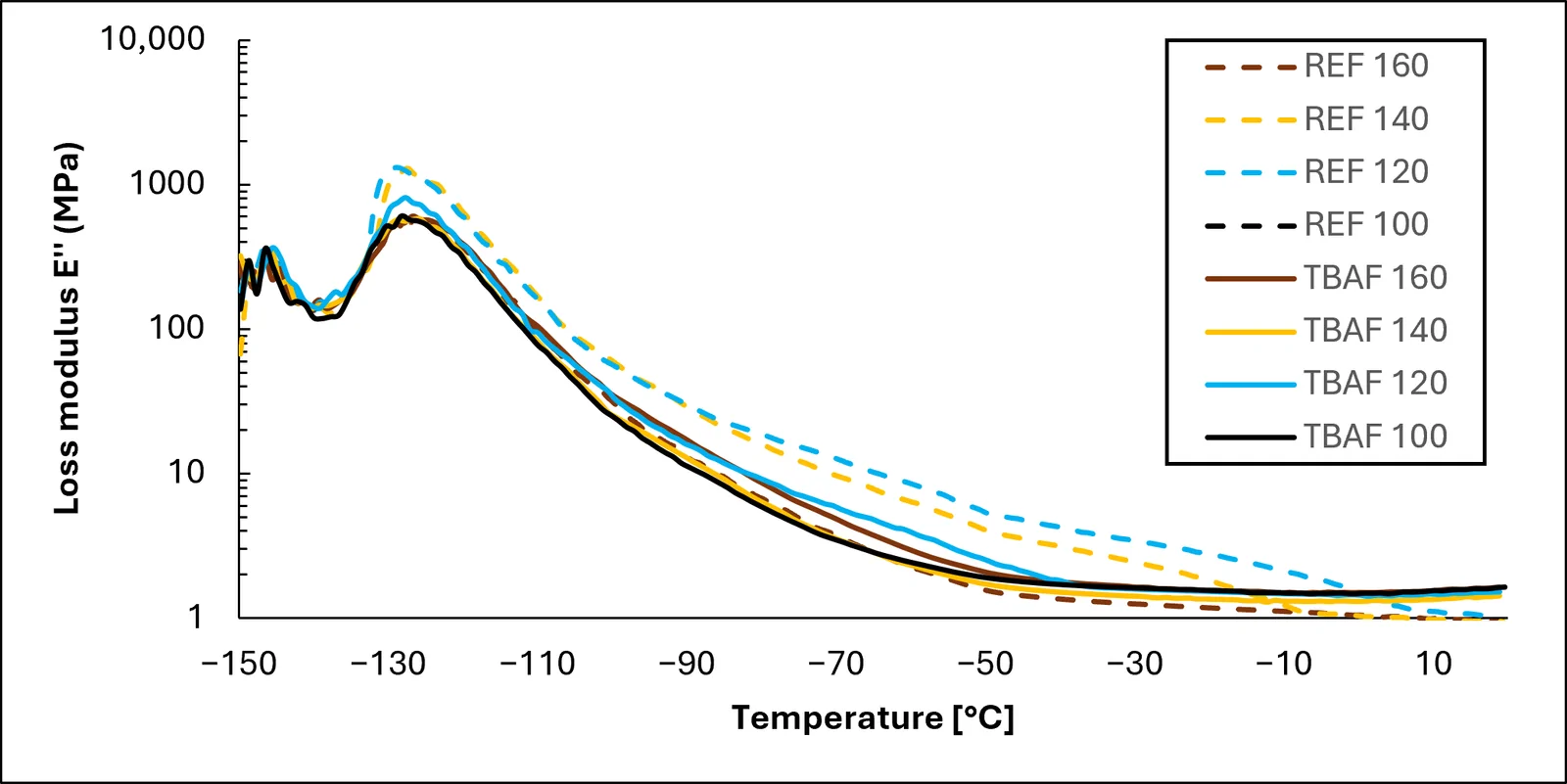
Loss modulus (E″) curves of the BR/VMQ composites vulcanized at different temperatures in the presence (TBAF) or absence (REF) of fluoride anion in the compound.

The storage modulus, loss modulus, and tanδ curves recorded between −150 °C and 20 °C are presented in Figure 5, Figure 6 and Figure 7, and the selected parameters (temperature of the loss modulus maximum T(E″max), temperature T(tanδ_max_) and height of the tanδ maximum, and tanδ values for 0 °C and −40 °C) are collected in Table 6.

All samples display glassy storage moduli of 8–10 GPa at −150 °C and a broad glass transition relaxation of the blend with tanδ maxima located between −115.3 °C and −117.3 °C, confirming that the vulcanizates remain in the rubbery state throughout the majority of the Martian surface temperature range. Two systematic differences between the series are evident. First, at every curing temperature, the TBAF vulcanizates exhibit lower tanδ peaks than the corresponding REF samples (0.85–1.15 vs. 0.94–1.38), i.e., more elastic, less dissipative properties in the transition region. Second, the relaxation of the TBAF series tends to shift slightly towards lower temperatures at the lowest curing temperatures: the E″ maxima of TBAF 120 and TBAF 100 (−127.7 °C and −128.2 °C) lie below those of TBAF 160 and TBAF 140 (−126.8 °C and −125.7 °C), and the tanδ maximum of TBAF 120 (−117.3 °C) is about one degree below those of the higher-temperature cures. If the tanδ (or E″) peak is read as the temperature of the glass transition, Tg of the TBAF vulcanizates therefore decreases slightly with decreasing curing temperature. While the changes in recorded peaks of E″ and tanδ are minor, they parallel the observed decrease in crosslink density: a looser network with long, flexible polysulfidic bridges imposes fewer constraints on segmental motion, allowing cooperative relaxation to occur at lower temperatures. Importantly, as shown in Section 3.5, this trend is not observed by DSC, where the calorimetric glass transition remains unchanged. The observed shift in tanδ peak reflects, therefore, network-related changes in the segmental dynamics measured under oscillatory load, rather than changes in calorimetric glass transition. No analogous monotonic shift is observed for the REF series, whose tanδ peak temperatures do not present a clear trend. The visible broad asymmetric tanδ peak, featuring an abrupt increasing slope on the low-temperature side and a slow, descending shoulder on the high-temperature side may come from the glass transition peak of the BR phase around −90 °C being overlapped by the VMQ relaxation due to macroscopic mechanical coupling of the blend.

The most application-relevant feature of the DMA results, however, lies above the transition region. All four TBAF vulcanizates exhibit a remarkably flat tanδ plateau extending from approximately −50 °C up to +20 °C: above −50 °C, their tanδ remains confined between 0.083 and 0.110 regardless of the curing temperature, with variations of less than ±0.01 over the range −40 °C to 0 °C (Table 6). The reference series does not share this stability: REF 140 and REF 120 still dissipate energy strongly at −40 °C (tanδ = 0.21 and 0.29, respectively), and their loss factors keep decreasing across the entire sub-zero range. To put this into a dynamic-performance context: the daily temperature amplitude on the Martian surface can reach 100 °C, with typical ranges from about +10 °C during the day down to about −90 °C at night [3,4], and Martian conditions may include temperatures as low as −120 °C [4]. The plateau of the TBAF vulcanizates therefore covers a major portion of the Martian daily cycle—roughly from the daily maximum down to −60 °C, within which both the stiffness and the damping of the material remain essentially constant. For a component such as a rover wheel or seal, this means that spring rates and hysteretic heat generation would not drift over most of a sol (Martian day), with significant changes confined to the coldest night-time hours. Furthermore, in the storage modulus (E′) curves (Figure 6), a drop in the modulus is visible at approximately −50 °C, which is likely an effect of the melting of the crystalline VMQ phase, further investigated by the DSC method (Section 3.5).

It is also worth noting that the rubbery plateau storage moduli of the TBAF vulcanizates (E′ ≈ 15–21 MPa at −20 °C) are consistently higher than those of the REF series (≈12–13 MPa), despite their lower chemical crosslink densities.

### 3.5. Differential Scanning Calorimetry

**Figure 8:**
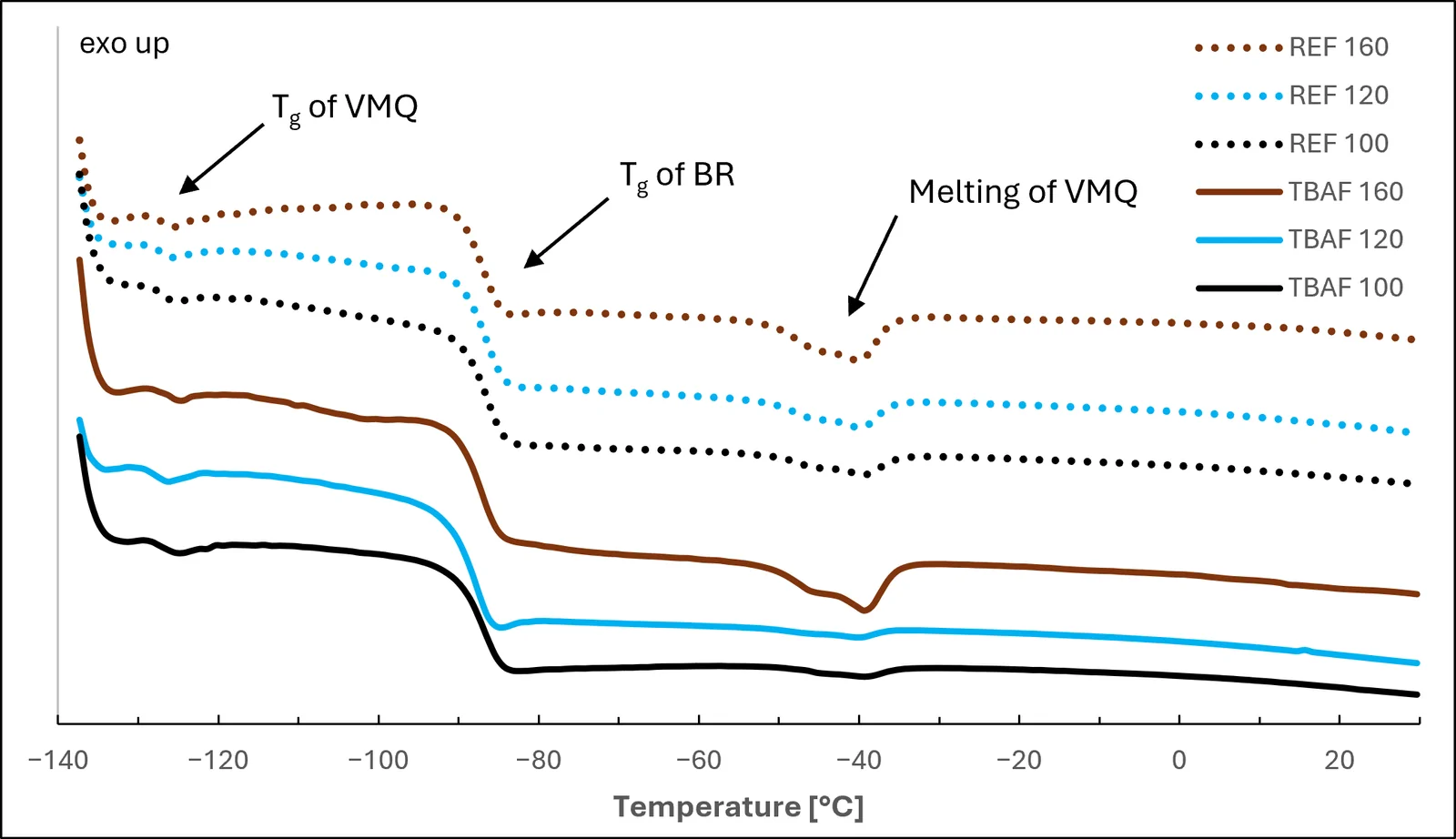
DSC heating curves (20 K/min) of the BR/VMQ vulcanizates cured at 160 °C, 120 °C, and 100 °C in the presence (TBAF) or absence (REF) of fluoride anion in the compound.

The DSC heating curves of the vulcanizates (Figure 8) show three features: a weak step near −125 °C attributable to the glass transition of the VMQ phase, the pronounced glass transition step of the BR-rich matrix near −90 °C, and an endothermic melting peak of the crystalline fraction of the VMQ phase centered at about −40 °C. The location of this endotherm agrees with the known melting of polydimethylsiloxane crystallites; PDMS is a crystalline polymer with a melting point near −40 °C and a glass transition temperature near −125 °C [26], and its crystallization on cooling and subsequent melting on heating are well documented in DSC studies [26]. The extracted parameters are given in Table 7. The medium-cis BR used in this study is amorphous [3]; accordingly, no BR melting endotherm is observed.

**Figure 9:**
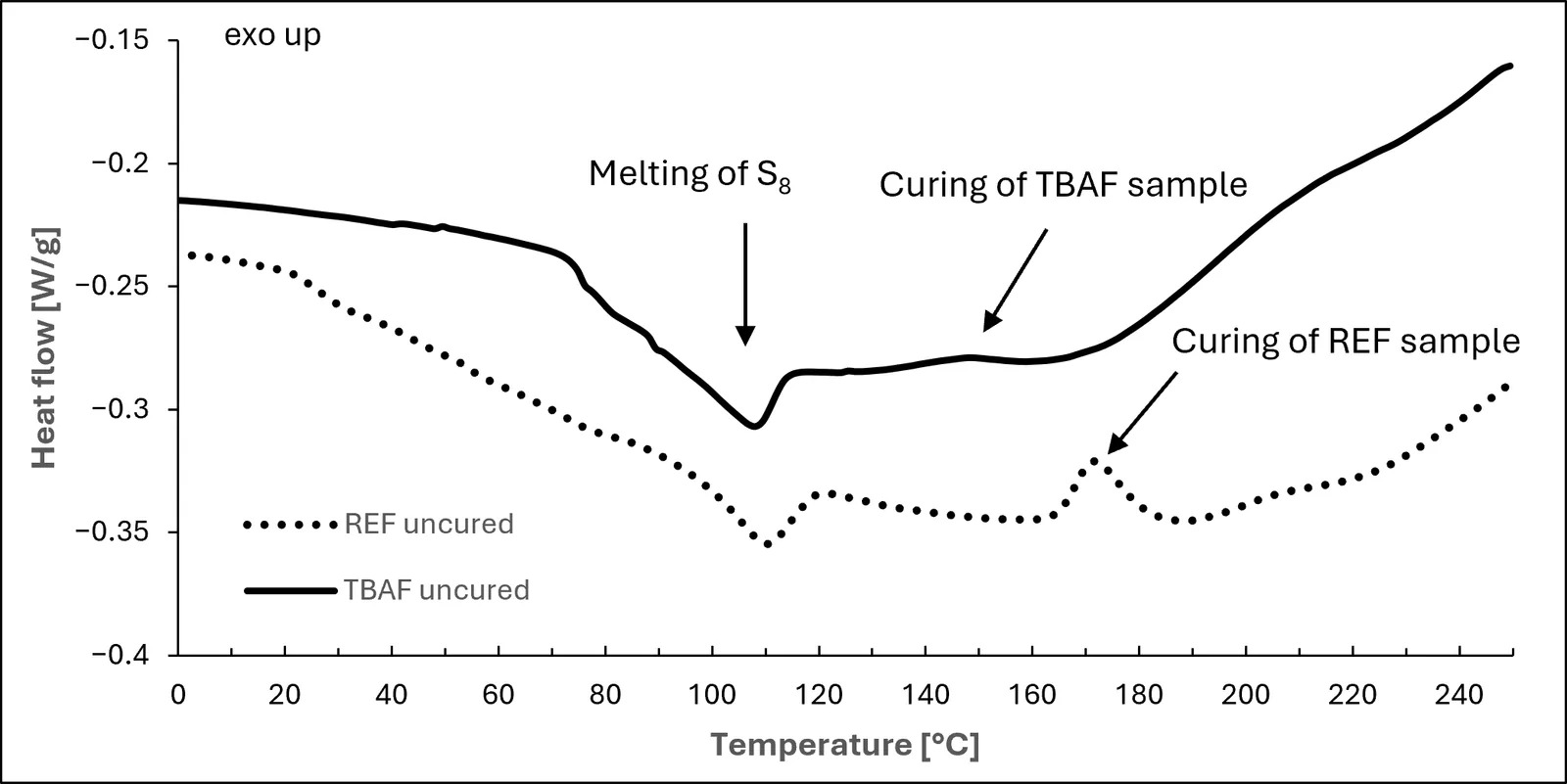
DSC plots showing the curing of the BR/VMQ composites in the presence (TBAF) or absence (REF) of fluoride anion in the compound.

The DSC measurements of uncured compounds are presented in Figure 9 and were discussed in Section 3.1. as they are relevant for the characteristics of vulcanization.

Two observations deserve emphasis. First, the calorimetric glass transition of the BR/VMQ matrix is independent of vulcanization temperature: the midpoints of Tg_BR_ of all six measured samples fall within the narrow interval from −86.9 °C to −88.6 °C, with no systematic dependence on either the curing temperature or the presence of TBAF. A similar situation is observed for Tg_VMQ_ where the midpoints of the glass transition fall in the range of −126.6 °C to −127.8 °C. This differs from the DMA results, where the mechanical relaxation of the TBAF series shifts slightly towards lower temperatures with decreasing curing temperature. The comparison shows that the chemical environment influencing the calorimetric Tg is unchanged, and that the DMA Tg shift originates from the changes in network architecture (lower crosslink density, longer sulfur bridges), influencing the segmental dynamics probed under oscillatory load, while leaving the calorimetric (heat-capacity-based) glass transition essentially unaffected.

Second, and more strikingly, the melting enthalpy of the VMQ phase drastically decreases when the vulcanization temperature is reduced. For the reference compounds, ΔHm decreases from 1.19 J/g (160 °C) to 0.51 J/g (100 °C), i.e., by about half; for the TBAF compounds the effect is far stronger, from 1.23 J/g at 160 °C down to 0.21 J/g and 0.25 J/g at 120 °C and 100 °C—a reduction of more than 80%—while the melting peak position itself (≈−40 °C) remains unchanged, indicating that the lamellae which do form are present in a much smaller amount. Since the BR grade used here (CB550) is amorphous [3], the entire crystallinity signal originates from the dispersed VMQ phase. We propose the following interpretation for this phenomenon: BR and VMQ are strongly immiscible, and the majority of phase separation occurs during the high-temperature vulcanization step, when chain mobility is at its maximum, and the still-developing network permits large-scale diffusion. When curing is performed at 160 °C, the VMQ chains have sufficient thermal energy and time to diffuse out from the BR-rich surroundings into separate domains, within which the highly regular dimethylsiloxane sequences can later pack into crystalline lamellae on cooling. When curing is performed at 100–120 °C, there is simply less energy available for this phase separation and for the local ordering of chain segments: interdiffused BR/VMQ interphases are locked in by the growing network before diffusion is complete, leaving VMQ chains partially constrained and unable to arrange into regular crystalline domains. The effect is strongest for the TBAF compounds because the fluoride-activated cure fixes the morphology particularly quickly (t_90_ of 16.9 min at 120 °C vs. 36.7 min for REF), stopping phase separation at an earlier stage and giving less time for the crosslink formation, which in itself can be an additional factor limiting chain mobility. Morphological verification of this hypothesis (e.g., by AFM or TEM) is planned as future work.

For the target application, the suppression of VMQ crystallinity is a positive side effect rather than a drawback. Melting/crystallization of the VMQ phase around −40 °C (exactly in the middle of the Martian operating window) introduces a first-order transition accompanied by volume and stiffness changes on every daily cycle. In the TBAF vulcanizates cured at 100–120 °C, this transition is drastically reduced, which is consistent with the exceptionally flat tanδ plateau and the storage modulus (E′) curves (where a drop in modulus is visible at approximately −50 °C) of these materials discussed in Section 3.4, and complements our earlier strategy of suppressing crystallization-related volume changes by selecting an amorphous BR [3].

## 4. Conclusions

BR/VMQ blends intended for Mars exploration applications were vulcanized at 160, 140, 120, and 100 °C using a conventional sulfur/CBS curing system (REF) and the same system activated with tetra-n-butylammonium fluoride (TBAF, patent no. PL 249368). The following conclusions can be drawn:

1. TBAF drastically accelerates low-temperature sulfur vulcanization, reducing the optimum cure time at 100 °C from 129.1 min to 61.4 min and at 120 °C from 36.7 min to 16.9 min, and lowering the DSC curing exotherm onset by approximately 31 °C. According to the earlier research, this is caused by the in situ generation of nucleophilic fluoropolysulfide anions that bypass the energy-intensive homolytic opening of the S_8_ ring [11,12,13].
2. Curing temperature steers the network architecture in opposite directions for the two systems. The reference networks gain crosslink density and shift to shorter crosslinks upon low-temperature/long-time curing (crosslink density rising from 1.25 × 10^−4^ to 1.56 × 10^−4^ mol/cm^3^, polysulfidic content falling from 89.6% to 84.1% between 160 °C and 120 °C), which we attribute to extended crosslink maturation. The TBAF networks, cured quickly and with less thermal energy, are frozen in an immature state of lower density (1.32 × 10^−4^ → 0.60 × 10^−4^ mol/cm^3^) dominated by elastic polysulfidic crosslinks (up to 97.2%).
3. The TBAF vulcanizates cured at 100–120 °C combine this polysulfidic architecture with extensibility at −40 °C, and exhibit lower tanδ maxima together with a flat tanδ plateau (≈0.08–0.11) extending from about −60 °C to +20 °C. Given that the Martian daily temperature amplitude reaches 100 °C (roughly +10 °C to −90 °C) [3,4], this plateau can potentially provide stable stiffness and damping over a very large portion of the daily cycle.
4. When observed as the tanδ (or E″) peak in DMA, the glass transition of the TBAF vulcanizates shifts slightly towards lower temperatures with decreasing curing temperature—tanδ maxima from about −116.3/−115.4 °C (TBAF 160/140) down to −117.3/−116.1 °C (TBAF 120/100)—following the decrease in crosslink density. No such trend appears in DSC, where the calorimetric Tg of BR of all samples remains at −87 to −89 °C, and the calorimetric Tg of VMQ of all samples remains at −127 to −128 °C. This observed behavior is consistent with network-related changes.
5. Low-temperature curing strongly suppresses crystallization of the VMQ phase: its melting enthalpy at ≈−40 °C drops from 1.23 J/g (TBAF 160) to 0.25 J/g (TBAF 100). We hypothesize that the rapid network formation at reduced curing temperatures restricts the separation of VMQ chains from the BR matrix, immobilizing the morphology before regular crystalline domains can fully develop. Further microscopic characterization is planned to verify this interpretation.

Overall, fluoride-activated low-temperature vulcanization offers a route to BR/VMQ materials that are potentially more energy-efficient to process, being cured 40–60 °C below conventional practice, and better suited to the Martian environment than their conventionally cured counterparts due to their broad and stable rubbery plateau in the region of the daily temperatures of Mars.

## Figures and Tables

**Table 1:** Formulations of the BR/VMQ compounds given in parts per hundred of rubber by weight (phr).

Component	REF	TBAF
BR (Buna CB550)	80	80
VMQ (Polimer MV 1,0)	20	20
Carbon black N330	30	30
Zinc oxide	3	3
Stearic acid	3	3
CBS	1.6	1.6
Sulfur	1.2	1.2
6PPD	2	2
TBAF	–	0.74

**Table 2:** Mixing procedure of the BR/VMQ compounds.

Time	Action	Rotor Speed [rpm]
0:00	Add rubbers, zinc oxide, stearic acid, 6PPD, and carbon black	30
3:00	Increase rotor speed	60
5:00	Decrease rotor speed, add CBS, TBAF (if applicable), and sulfur	30
5:30	Increase rotor speed	60
6:30	Stop mixing, unload the mixer	0

**Table 4:** Crosslink density (ν) and crosslink structure of the BR/VMQ vulcanizates. Samples marked with an asterisk (*) crumbled after the selective degradation of di- and polysulfidic bonds. Crosslink density values are presented as mean ± standard deviation (*n* = 4).

Sample	ν [mol/cm^3^]	Polysulfidic (S_x_) [%]	Disulfidic (S_2_) [%]	C–C + Monosulfidic [%]
REF 160	(1.25 ± 0.04) × 10^−4^	89.6	8.4	2.0
REF 140 *	(1.38 ± 0.07) × 10^−4^	87.5	12.5	0
REF 120	(1.56 ± 0.05) × 10^−4^	84.1	15.2	0.7
REF 100 *	(1.42 ± 0.06) × 10^−4^	96.9	3.1	0
TBAF 160	(1.32 ± 0.03) × 10^−4^	72.6	25.8	1.6
TBAF 140 *	(1.10 ± 0.04) × 10^−4^	87.8	12.2	0
TBAF 120 *	(0.78 ± 0.19) × 10^−4^	97.2	2.8	0
TBAF 100 *	(0.60 ± 0.04) × 10^−4^	96.5	3.5	0

**Table 5:** Tensile strength (T_S_) or stress at maximum elongation T_Fmax_ (indicated with †), elongation at break (E_B_) values for BR/VMQ vulcanizates obtained at room temperature and at −40 °C. Values are presented as mean ± standard deviation (*n* = 4).

Sample	−40 °C		Room Temperature	
	T_S_ or T_Fmax_ (†) [MPa]	E_B_ [%]	T_S_ [MPa]	E_B_ [%]
REF 160	16.6 ± 0.5 †	>1000%	4.9 ± 0.4	530 ± 40
REF 140	15.1 ± 0.3	906 ± 13	5.4 ± 0.2	550 ± 10
REF 120	14.2 ± 1.2	860 ± 42	5.7 ± 0.2	505 ± 10
REF 100	16.3 ± 0.2 †	>1000%	8.5 ± 0.1	745 ± 18
TBAF 160	7.3 ± 0.9	597 ± 53	4.6 ± 0.5	381 ± 36
TBAF 140	13.9 ± 1.5	883 ± 53	5.1 ± 0.3	506 ± 15
TBAF 120	13.9 ± 0.7 †	>1000%	5.8 ± 0.2	626 ± 9
TBAF 100	14.3 ± 0.1 †	>1000%	6.3 ± 0.1	662 ± 10

**Table 6:** DMA parameters of the BR/VMQ vulcanizates: temperature of the loss modulus maximum T(E″max), temperature T(tanδ_max_) and height of the tanδ maximum, and tanδ values within the plateau region.

Sample	T(E″max) [°C]	T(tanδ_max_) [°C]	tanδ_max_ [–]	tanδ at −40 °C [–]	tanδ at 0 °C [–]
REF 160	−125.7	−116.3	0.94	0.101	0.093
REF 140	−127.4	−115.9	1.37	0.210	0.086
REF 120	−128.8	−115.3	1.38	0.285	0.127
REF 100	−127.2	−115.7	1.27	0.126	0.098
TBAF 160	−126.8	−116.4	0.85	0.083	0.085
TBAF 140	−125.7	−116.3	0.96	0.083	0.086
TBAF 120	−127.7	−117.3	1.15	0.094	0.090
TBAF 100	−128.2	−116.1	0.96	0.084	0.088

**Table 7:** DSC parameters of the BR/VMQ vulcanizates: glass transition midpoints of the VMQ phase (Tg_VMQ_) and of the BR phase (Tg_BR_), melting peak temperature (T_m_), and melting enthalpy (ΔHm) of the VMQ phase (heating rate 20 K/min). Note: The ΔH_m_ values represent apparent melting enthalpies normalized to the total mass of the composite, rather than per gram of VMQ.

Sample	Tg_VMQ_ [°C]	Tg_BR_ [°C]	T_m_ (VMQ) [°C]	ΔH_m_ (VMQ) [J/g]
REF 160	−127.4	−87.4	−40.2	1.19
REF 120	−127.8	−86.9	−39.9	0.74
REF 100	−127.6	−86.9	−39.6	0.51
TBAF 160	−126.7	−87.6	−39.2	1.23
TBAF 120	−127.8	−88.6	−40.0	0.21
TBAF 100	−126.6	−87.4	−39.6	0.25

## Data Availability

The original data presented in the study are openly available in the ResearchGate profile of the first author at https://doi.org/10.13140/RG.2.2.28100.80007.
